# The Endophytic Strain *Klebsiella michiganensis* Kd70 Lacks Pathogenic Island-Like Regions in Its Genome and Is Incapable of Infecting the Urinary Tract in Mice

**DOI:** 10.3389/fmicb.2018.01548

**Published:** 2018-07-16

**Authors:** Karina I. Dantur, Nadia R. Chalfoun, Maria P. Claps, Maria L. Tórtora, Clara Silva, Ángela Jure, Norma Porcel, Maria I. Bianco, Adrián Vojnov, Atilio P. Castagnaro, Björn Welin

**Affiliations:** ^1^Instituto de Tecnología Agroindustrial del Noroeste Argentino, Estación Experimental Agroindustrial Obispo Colombres – Consejo Nacional de Investigaciones Científicas y Técnicas, Las Talitas, Argentina; ^2^Instituto de Microbiología, Facultad de Bioquímica, Química y Farmacia, Universidad Nacional de Tucumán, Tucumán, Argentina; ^3^Instituto de Ciencia y Tecnología Dr. César Milstein, Fundación Pablo Cassará – Consejo Nacional de Investigaciones Científicas y Técnicas, Buenos Aires, Argentina

**Keywords:** draft genome, plant growth promotion, sugarcane, genomic analysis, virulence

## Abstract

*Klebsiella* spp. have been isolated from many different environmental habitats but have mainly been associated with nosocomial acquired diseases in humans. Although there are many recently published sequenced genomes of members of this genus, there are very few studies on whole genome comparisons between clinical and non-clinical isolates, and it is therefore still an open question if a strain found in nature is capable of infecting humans/animals. *Klebsiella michiganensis* Kd70 was isolated from the intestine of larvae of *Diatraea saccharalis* but genome analysis revealed multiple genes associated with colonization and growth promotion in plants suggesting an endophytic lifestyle. Kd70 cells labeled with *gfp* confirmed capability of root colonization and soil application of Kd70 promoted growth in greenhouse grown sugarcane. Further genomic analysis showed that the Kd70 genome harbored fewer mammalian virulence factors and no pathogen island-like regions when compared to clinical isolates of this species, suggesting attenuated animal/human pathogenicity. This postulation was corroborated by *in vivo* experiments in which it was demonstrated that Kd70 was unable to infect the mouse urinary tract. This is to the best of our knowledge the first experimental example of a member of a pathogenic *Klebsiella* spp. unable to infect a mammalian organism. A proteomic comparison deduced from the genomic sequence between Kd70 and several other *K. michiganensis* strains showed a high similarity with isolates from many different environments including clinical strains, and demonstrated the existence of conserved genetic lineages within this species harboring members from different ecological niches and geographical locations. Furthermore, most genetic differences were found to be associated with genomic islands of clinical isolates, suggesting that evolutionary adaptation of animal pathogenicity to a large extent has depended on horizontal gene transfer. In conclusion our results demonstrate the importance of conducting thorough *in vivo* pathogenicity studies before presupposing animal/human virulence of non-clinical bacterial isolates.

## Introduction

*Klebsiella* is a genus of Gram-negative, rod-shaped, non-motile, facultative anaerobe bacteria that normally produces a prominent polysaccharide capsule which provides resistance against host defense systems. *Klebsiella* spp. have been isolated and characterized in many different environmental habitats including soil, food, plants, insects, and water but have mainly been associated with nosocomial acquired diseases in humans (Brisse et al., 2006) with a longstanding association with hospital acquired pneumonia, blood stream, and urinary tract infections (Peleg and Hooper, 2010; Foxman, 2014). The spectrum of infections associated with *Klebsiella* spp. is, however, changing, involving community-acquired infections (Högenauer et al., 2006), and other types of infectious diseases (Janda, 2015). Although members of this genus have predominantly been studied in association with human and animal pathogenicity there are numerous studies where *Klebsiella* spp. have been shown to enhance plant’s growth by different mechanisms, although the exact basis for this stimulation is not completely resolved.

One of the principal hypothesis explaining the plant growth promotion capacity of *Klebsiella* spp., has been the capacity to fix atmospheric nitrogen when associated with gramineous species (Vessey, 2003; Temme et al., 2012; Zhu et al., 2012; Bao et al., 2013; Mingyue et al., 2013). However, *Klebsiella* spp. are also able to produce high quantities of extracellular phytohormones like indoleacetic acid (IAA) and tryptophol and low levels of indolepyruvate and indoleacetaldehyde (El-Khawas and Adachi, 1999; Shokri and Emtiazi, 2010; Liu et al., 2013), to solubilize phosphate by expressing a significant level of acid phosphatases (Thaller et al., 1995; Ahemad and Kibret, 2014) and to produce catechol and hydroxamate types of siderophores (Podschun et al., 1992, 2001), traits that are strongly associated with plant growth promotion by providing the plant with nutrients, minerals, and hormones (Glick, 2012). Another important characteristic found for many PGPR strains including *Klebsiella* spp. is their capacity to form biofilm. The formation of biofilm is important for effective colonization on or in the plant root, helping the bacteria to compete well with indigenous microflora and at the same time generating improved plant growth promotion by improved N_2_-fixation, mineral uptake, phosphorus solubilization or abiotic and pathogen protection (Seneviratne et al., 2011).

Bacterial strain Kd70 was isolated from the intestine of larvae of the moth *Diatraea saccharalis* feeding on sugarcane (Dantur et al., 2015), indicating a possible endophytic origin of this bacteria. The isolation of Kd70 was part of a search for lignocellulose-degrading bacteria in order to isolate cellulases and hemicellulases efficient in degradation of sugarcane biomass. It is well-known that endophytic bacteria need to be equipped with cellulolytic and pectinolytic enzymes in order to hydrolyze the plant’s exodermal cell walls for active penetration (Compant et al., 2005, 2010). This coincides with the documented plant cell wall degrading activity found for strain Kd70 and has previously been demonstrated in other *Klebsiella* strains able to colonize plant roots (Kovtunovych et al., 1999; Yu et al., 2018).

Kd70 was initially described as a member of the species *Klebsiella oxytoca* but has recently been recategorized as *K*. *michiganensis* (Saha et al., 2013), together with numerous other strains previously described as *K*. *oxytoca*, based on the average nucleotide identity (ANI) approach, calculated from pair-wise comparisons of sequences shared between close relatives (Konstantinidis et al., 2006; Tindall et al., 2010).

The sequencing of the genome of Kd70 allowed us to gather genetic information on the ability of this strain to promote plant growth and development, but also to study genetic differences among strains isolated from different ecological niches, which could help to shed light on the great variance of ecotypes and lifestyles found among members belonging to this genus. As expected the Kd70 genome harbors a vast variety of plant growth promoting genes associated with nitrogen fixation, IAA synthesis, phosphate solubilization and siderophores production indicating that this strain is indeed an important growth promoting endophyte in sugarcane. Comparing the genome of Kd70 with genomes of other *K. oxytoca*/*michiganensis* strains isolated from clinical habitats showed that Kd70 encodes fewer animal/human virulence factors and possess less genes involved in antibiotic resistance as compared to clinical isolates from humans and animals. This finding indicated an attenuated animal virulence for this strain, an observation that was confirmed by ascending urinary tract infection experiments in mice.

## Materials and Methods

### Bacterial Strains and Growth Conditions

The bacterial strains used in this work was *K. michiganensis/oxytoca* Kd70 (Dantur et al., 2015), *K. michiganensis/oxytoca gfp*-Kd70, *Pseudomonas fluorescent* P21 (unpublished own isolate), *P. syringae* 23 (unpublished own isolate), and *Bacillus pumilis* Kd101 (unpublished own isolate). Pure colonies of bacterial isolates were cultivated routinely in LB broth or minimal saline agar medium (Dantur et al., 2015) containing 0.2% of glucose and 0.2% tryptone, incubated at 37°C for 16 h and appropriately diluted before use.

### DNA Extraction, Whole-Genome Shotgun Sequencing, and Genome Sequence Analysis

DNA extraction was performed using the PureLink Genomic DNA Kit (Invitrogen, United States) according to the instruction of the manufacturer. The draft sequence of the complete genome of strain *K. michiganensis/oxytoca* Kd70 (DSM27019) was obtained by preparing total bacterial DNA by the 454 sequence preparation kit (Roche Applied Sciences, Indianapolis, IN, United States) and subsequent sequencing using the GS-FLX Titanium technology (454 Life Sciences, Roche Applied Sciences, United States). All 454 reads were assembled using software Newbler 2.6. Overall sequence coverage was 40×. Sequence annotation was performed using RAST Server software (Aziz et al., 2008) followed by manual inspection.

### Nucleotide Sequence Accession Number

The whole-genome shotgun project of *K. michiganensis/oxytoca* Kd70 has been deposited at GenBank under the accession number LGRU00000000. The version presented in this paper is the first version, LGRU01000000. Data sets are accessible through NCBI’s sequence read archive (SRA), under study accession number SRP [061608].

### Mapping Contigs

Of the total number of contigs generated from the sequencing of the Kd70 genome, 40 was assembled by mapping against two related reference genomes of *K. michiganensis/oxytoca* strains JKo3 and E718, using the CONTIGuator algorithm version 2.7.4 (Galardini et al., 2011, 2015).

### Bioinformatic and Phylogenetic Analysis

Prediction of protein encoding sequences and open reading frames (ORFs) was conducted by comparing protein sequences with the public databases SwissProt, GenBank, KEGG, COG, and Prosite. For further comparative genomic analysis, the generated pseudocontig of Kd70 was standardized using RAST annotation at PathoSystems Resource Integration Center (PATRIC) (Snyder et al., 2007; Gillespie et al., 2011). The set of genes related to virulence and potential antibiotic resistance development were identified and the Proteome Comparison tool allowed for examining the degree of protein similarity among *K. michiganensis*/*oxytoca* genomes, using the Kd70 pseudogenome as reference.

To predict genomic islands (GIs) IslandPick, IslandPath-DIMOB, SIGI-HMM, and Islander methods, integrated into the recently single web platform IslandViewer 4^1^ (Bertelli et al., 2017), was used. The same platform was used to identify pathogen-associated genes (Liu and Pop, 2009; Chen et al., 2012) and possible pathogenic islands. Comparison of GIs and the set of genes forming GIs between the Kd70 and E718 and JKo3 strains were performed by crossing data from the platforms PATRIC, IslandViewer 4, and NCBI BLAST.

For phylogenetic analysis the PEPR software, provided by the PATRIC platform, making use of several third-party tools (BLAST, MCL, Muscle, hmmbuild, hmmsearch, and Gblocks), and the tree building application RAxML (Stamatakis, 2014) with automated progressive refinement was used for whole genome comparisons of *K. michiganensis*/*oxytoca* isolates.

### Siderophore Production

Siderophores were detected by using the universal chrome azurol sulfonate (CAS, Sigma–Aldrich, United States) assay (Schwyn and Neilands, 1987) visualizing color change of the CAS-iron complex (from blue to orange) after iron chelation by siderophores. The CAS agar plate [60.5 mg CAS dissolved in 50 ml deionized water and mixed with 10 ml iron(III) solution (1 mM FeCl_3_ 6H_2_O in 10 m MHCl)]. Five microliter of bacterial suspensions were spotted on CAS plates and incubated 7 days at 30°C. A time course of siderophore production was monitored by the formation of an orange halo around the colonies. Siderophore production yield (%Ys) was determined as [(halo diameter-colony diameter)/colony diameter] × 100 and *Pseudomonas fluorescens* strain P23 was used as a positive control. Each assay was performed in triplicate and results were subjected to analysis of variance (ANOVA) and LSD (*P* = 0.05) analysis by using the Statistix Analytical Software 1996 for Windows (United States).

To study the effect of iron concentration on bacterial siderophore production, 50 or 100 μM FeCl_3_.6H_2_O was added to agar CAS plates later inoculated with bacterial suspensions.

### Phosphate Solubilizing Assay

Phosphate solubilizing was tested using National Botanical Research Institute’s phosphate (NBRIP) medium (Nautiyal, 1999). Plates inoculated with 5 μl of an overnight bacterial culture were incubated at 30°C and observed daily for formation of transparent halos around each colony during 7 days. The ability of the bacteria to solubilize insoluble phosphate was defined by the solubilization index [(halo diameter + colony diameter)/colony diameter] (Edi Premono et al., 1996). *P. fluorescens* strain P21 was used as positive control. Each assay was performed in triplicate and statistically analyzed as described for siderophore production.

### Screening for Biofilm Formation

Biofilm formation was detected using the crystal violet (CV) method (Christensen et al., 1985). Bacteria at stationary growth phase was inoculated in 96-microwell plates containing 200 μl of LB medium, until reaching an OD_495_ of 0.2 and incubated for 6 days at 30°C without agitation. After 6 days of incubation the supernatant was removed and plates were washed twice with 0.9% NaCl (w/v). For staining, 200 μl of a 0.1% CV (w/v) solution was added per well and incubated for 15 min. The excess of CV was removed by washing plates three times with 0.9% NaCl (w/v) and bound CV was released from cells by adding 200 μl of 95% EtOH, where after absorbance of the suspension was measured at 595 nm. Data were normalized by total growth estimated by measuring the absorbance at 495 nm. Bacterial strains *Pseudomonas* spp. P21, known as a good biofilm producer and *Bacillus pumilus* Kd101, a very weak biofilm producer, were used as internal controls. Culture media without bacteria was used as a negative biofilm control. Each assay was performed in triplicate and statistically analyzed as described for siderophore production.

### Total Production of Indoles

Total concentration of indoles was determined by a colorimetric method (Glickmann and Dessaux, 1995) using a minimum saline medium (MSM) supplemented or not with tryptophan (Trp) (0.1 mg/ml). Bacteria was streaked out on solid LB medium and incubated at 30°C overnight, a single colony was thereafter transferred to liquid MSM. After 7 days growth at 30°C, total indole production was evaluated by mixing 500 ml of the supernatant with 500 ml of Salkowski reagent, followed by incubation in the dark at room temperature during 30 min. Total indoles were determined spectrophotometrically at 540 nm, using synthetic IAA as standard. The test was carried out in triplicate and statistical analyzed as described for siderophore production. Data were normalized according to total protein concentration of the supernatant as determined by the Bradford method (Bradford, 1976) using bovine serum albumin for the standard curve.

### Fluorescence Microscopy

Strain Kd70 was transformed by conjugation as previously described (Simon, 1984) using *Escherichia coli* S17-1 carrying the plasmid pBBR2-GFP to generate strain *gfp-*Kd70. Plasmid pBBR2-GFP is a derivative of pBBR1MCS-2 (Kovach et al., 1994, 1995) and harbors the gene encoding the green fluorescent protein (GFP), regulated by a constitutive bacterial promoter (Posadas et al., 2012).

One-month-old *in vitro* propagated plantlets were co-cultured with *gfp*-Kd70 cells adjusted at 1.0 × 10^8^ cells/ml for 7 days. Sterile water was used as a non-inoculated control. Plantlets were thereafter transferred to autoclaved sand and perlome mixtures and grown for 20 days *ex vitro*. Hand-cut transverse sections of roots inoculated with either water (control) or the *gfp*-Kd70 strain were observed under UV light in a fluorescence microscope (BX51 U-LH 100HG, Olympus, Germany) to visualize root colonization by *gfp-*Kd70.

### Plant Growth Promotion Assay in Greenhouse-Grown Sugarcane

*In vitro* micropropagated sugarcane plants (Lee, 1987; Basnet et al., 2007) of the commercial variety LCP 85-384 were used to evaluate a possible plant growth promoting effect of strain Kd70.

Kd70 was cultivated in adaptation media (Dantur et al., 2015) o/n at 30°C at 200 rpm. A commercial liquid biofertilizer (Gramen), based on bacterial strain *Azospirillum brasilense* Az39, with proven growth promotion activity in sugarcane, was used as a positive growth promotion control.

Two-months-old micropropagated plantlets, selected for similar plant size and root development, were submerged in a liquid fungicide solution [captan 0.2% (w/v)] during 12 h. Plantlets were thereafter washed with deionized water, individualized and planted in seedling trays containing a non-sterile substrate of soil-perlome 1:1 (w/w). Different soil characteristics such as organic matter (OM), available phosphorous, pH, salinity, carbonate, and texture were evaluated (see below). Once plantlets had rooted (after approximately 20 days), they were transferred to 4 l plastic pots filled with the same substrate. Plants were placed in a greenhouse with a temperature of 30°C and 95% RH and pots were placed in a completely randomized design with five replications for each treatment and five individuals for each replication. Treatments were as follow: (i) plants inoculated with Kd70; (ii) plants inoculated with Gramen; and (iii) plants treated with water as normal growth control. Bacterial inoculations were performed at three different time points. The first inoculation was done by complete immersion of micropropagated plantlets in a bacterial suspension for 30 min after fungicide treatment and immediately before plantation in seedling trays for rustication. Second and third inoculations were performed by watering plants with 200 ml of a bacterial suspension (10^6^ CFU/ml) 20 and 50 days after transfer to the 4 l plastic pot.

Tiller number dynamics and plant height from the soil surface up to the auricle region of the +1 leaf, were evaluated every 15 days after transplantation (dat) to larger pots. At the end of the assay, 60 dat, stem diameter, fresh, and dry weight of both plant aerial and root system were measured.

### Soil Physical Chemical Characteristics

Reference soil texture used for bioassays was silty loam with 2.4% of OM, which correlates with typical contents for sugarcane agricultural soils in this region. The pH soil value was 6.3 (1:2.5 mixture of dry soil and distilled water) determined using a combination glass electrode. Soil salinity was 0.2 dS/m according to the electrical conductivity of the saturated paste extract (CEe) at 25°C. Soil available phosphorus was 18 ppm and it did not contain any free calcium carbonate.

Results were analyzed with the statistical software InfoStat (Di Rienzo et al., 2011, 2014). LSD Fisher test was used to determine the arithmetic mean and the ANOVA test was used to evaluate data dispersion with respect to the mean value.

### Detection of Kd70 in Sugarcane Plant Tissues Using Species-Specific PCR Primers

Kd70-specific oligonucleotide primers were used to evaluate the successful root colonization of Kd70 in sugarcane by polymerase chain reaction (PCR) DNA amplification. Two pairs of primers were designed for two-independent genes involved in cellulolytic activity of Kd70, *Cbm*6-*Cbm*3, and *GH*3, according to genetic information obtained from the whole genome sequence of Kd70 and on the basis of sequence alignments of both genes to public sequence data bases. To confirm specificity of selected primers experimentally, DNA from several Gram-negative and Gram-positive bacteria, including other *K. michiganensis* strains, was used as template for PCR reactions. As an internal positive control for bacterial DNA amplification from plant tissue, a conserved sequence segment of the 16S ribosomal DNA was used. All PCR amplification primers used are described in the **Table 1** below.

**Table 1:** PCR amplification primers.

Genes	Primers
CBM6-CBM3	F 5′ AACGTGATTAGCGTGAAGCACACCC 3′
	R 5′ GGCGCTAAAGGTCAGCGCATATTCAC 3′
GH3	F 5′ TCTCATACACCCGCTTCACCCTGTC 3′
	R 5′ CCCGCTATCGTCCAAAGACGCAAAAC 3′
16S rRNA-70	F 5′ GGAATATTGCACAATGGGCGCAAGC 3′
	R 5′ TGCCAGTTTCGAATGCAGTTCCCAG 3′

Briefly, 10 g of roots from each plant were rinsed thoroughly with sterile water, surface disinfected by immersion in 4% sodium hypochlorite during 10 min and subjected to an extensive washing with sterile water. The surface-disinfected root material were grounded in a mortar and plated on solid CMC saline media in Petri dishes and incubated overnight at 37°C. Bacterial colonies able to grow on the cellulose media were selected for PCR reactions together with the total bacterial pool obtained directly from the macerated material. PCR was performed in a Bio-Rad Mycycler Thermalcycler (Hercules, CA, United States). Each reaction mixture (25 μl) contained 1× reaction buffer, 2 mM MgCl_2_, 0.2 μM of each primer, 0.2 mM of dNTPs, 2 U of Taq DNA polymerase (Invitrogen, United States), and 1 μl of genomic DNA. PCR cycling parameters were 1 cycle at 95°C (2 min); 35 cycles at 95°C (30 s), 54°C (30 s), and 72°C (3 min); and a final cycle at 72°C for 10 min. All amplified DNA products were checked by gel electrophoresis in 2% (w/v) agarose gels stained with GelRed (Biotium, United States).

### Antibiotic Resistance Test

The antimicrobial susceptibility pattern was established with the disk diffusion method and interpreted as specified by the M100-S24 Clinical and Laboratory Standards Institute (CLSI) (CLSI, 2014) document. The quality control was performed using the *E. coli* ATCC 25922, *E. coli* ATCC 35218, and *K. pneumoniae* ATCC 700603 reference strains. The following antibiotics were tested: ampicillin, cephalothin, cefotaxime, ceftazidime, cefepime, aztreonam, imipenem, meropenem, ciprofloxacin, trimethoprim plus sulfamethoxazole, gentamicin, and amikacin. Minimal inhibitory concentrations (MICs) of β-lactams, aminoglycosides, and fluorquinolones were determined with the *E*-test method (Biomérieux).

### Virulence Test

Uropathogenic *Escherichia coli* (UPEC) strain 275 isolated from an adult female patient with a diagnosis of pyelonephritis (from the Collection of Culture Strains of the Bacteriology Department, Instituto de Microbiologia of the Universidad Nacional de Tucumán, Argentina) was used as infective prototype (Silva et al., 2004).

Two-months-old female BALB/c mice from the Instituto de Microbiologia at the Universidad Nacional de Tucumán, Argentina, were used. All mice used for the experiment came from the same inbred, closed colony and of the same age and weight, to avoid genetic interference. All the control groups were assayed at the same time.

Each experimental group included 20–25 animals weighing 25–30 g, housed in plastic cages and fed *ad libitum* with a conventional balanced diet and keeping the environmental conditions constant.

All animals used in the experiment were synchronized inducing a pseudo-estrous condition with a subcutaneous single dose of 0.02 mg of β-estradiol 17-valerato (Sigma–Aldrich, Italy) dissolved in 100 μl sesame oil (Sigma) (Silva et al., 2004; Rahman et al., 2007; De Gregorio et al., 2014) every 5 days, to favor bacterial colonization. Forty eight hours after hormone administration mice were intravaginally inoculated with 50 μl of a 1 × 10^8^ CFU/ml E. coli 275 or Kd70 bacterial solution. Saline solution was administrated as a non-infective control. At 1, 2, 5, and 14 days after bacterial inoculation, the animals were sacrificed by cervical dislocation. The number of viable pathogen cells in asceptical vagina washes and homogenates of urethra, bladder, ureter, and kidney, was determined. Serial 10-fold dilutions from vagina exudates and urinary tract homogenates were quantified using ChromID ESBL agar (Biomérieux) (Paterson and Bonomo, 2005; Glupczynski et al., 2007). The cytological evaluation of vagina exudates stained with Giemsa, was carried out by light microscopy (AxioScope A1 Carl Zeiss). The experimental protocols were approved by the Ethical Committee of Animal Care of Reference Centre for Lactobacilli (CERELA-CONICET, Tucumán, Argentina), all efforts were made to minimize suffering (CERELA-CONICET^2^).

Each experimental protocol was independently repeated three times using three animals per group, two control animals inoculated with the pathogen and one without inoculating as a hormone control at each sampling time.

## Results

### General Features of the Kd70 Draft Genome

The draft genome of Kd70 consists of 54 contigs generating a total of 5,681,945 base pairs (bp), a G + C content of 56% and no extra chromosomal DNA. Gene prediction revealed 5,449 coding sequences (CDS) including 80 tRNA genes and 10 ribosomal RNA genes. Among the CDS, 4,792 (87.9%) were appointed as proteins with functional assignments and 657 (12.1%) as hypothetical proteins or proteins with unknown function.

The draft genome sequence of Kd70 was aligned against two previously sequenced clinical isolates of *K*. *oxytoca/michiganensis*, JKo3 one of its closest phylogenetic relatives and the genetically more distant E718, which were used as reference genomes. The contigs align software CONTIGuator mapped 33 contigs from the Kd70 sequencing (5,663,084 bp) to the reference genomes, which roughly corresponded to 99.66% of the total input nucleotide sequences. Only 0.34% was unmapped contigs (13,957 bp) and discarded due to poor coverage (1,763 bp), duplicated hits (5,843 bp) or short contigs (6,351 bp).

### Genomic Analysis

#### Genes Putatively Involved in Plant Growth Promotion

In-depth sequence analysis of the Kd70 genome revealed the presence of 318 ORFs (6%) encoding proteins putatively involved in plant growth promotion, which is in accordance with the presumed endophytic lifestyle of Kd70 (Supplementary Figure S1). These ORFs include genes encoding proteins involved in improving organic/inorganic nutrients availability to the plant, plant growth stimulation and development and suppression of plant pathogenic microbes.

More specifically, the Kd70 genome harbors a complete nitrogen fixation regulon (*nif*), 7 operons related to ammonia uptake, 25 genes encoding putative proteins related to nitrate/nitrite assimilation and metabolism and 11 ORFs related to urea uptake and conversion to ammonia including 1 operon comprising 7 urease coding genes and 1 operon formed by 4 urea-ABC-transporter genes.

Over 60 CDS were assigned to iron uptake and metabolism including genes encoding proteins with high resemblance to yersiniabactin/enterobactin siderophore biosynthesis, siderophore uptake, siderophore piracy receptors and CDS probably involved in ferrous and ferric iron siderophore-independent transport. Two other large groups involved in nutritional availability are genes encoding proteins related with inorganic sulfur assimilation and responsible for phosphate and phosphonate solubilization and uptake.

In addition to the above mentioned genes indirectly involved in plant growth, genes belonging to biosynthesis of the plant growth regulator auxin, IAA, with direct impact on plant development were identified.

#### Bacterial Colonization of Plant Roots

To further support the endophytic lifestyle of Kd70 we found compelling evidence in the form of putative adhesion and colonization proteins that could help the bacteria adhere to the plant root surface. Examples of such proteins encoded in the Kd70 genome are two putative hemagglutinin-related genes, 29 ORFs of Type I pili (mannose-sensitive fimbriae, Fim A–I), 10 genes encoding Type IV pili putatively involved in gliding/twitching motility and 2 site-specific recombinase genes that could be regulating fimbrae synthesis (Wu et al., 2010). Other genes potentially participating in the regulation and establishment of bacterial cells inside de plant through development of biofilm are exopolysaccharides components (6 genes) and genes encoding determinants involved in chemotaxis (*che*A), quorum sensing (10 genes), and Type VI secretion system (14 genes). Interestingly, Kd70 do not encode Type III fimbrae which has been associated with human and animal pathogenicity in other isolates of *Klebsiella* spp. (Tarkkanen et al., 1997; Schroll et al., 2010).

Finally, a total of 73 genes involved in carbohydrate metabolism, including 10 for cellulose hydrolysis, 15 for hemicellulase metabolism, 1 laccase, 37 encoding putative sugar transporters and utilization proteins and 10 related to pectin degrading enzymes (4 polysaccharide lyases and 6 carbohydrate esterases) were found. This is in agreement with our previous study showing that Kd70 is able to hydrolyze and grow on both cellulose and hemicelluloses (Dantur et al., 2015). Additionally, cellulases and hemicellulases have been considered as important activities for active entry of endophytes in plant tissue (Compant et al., 2005, 2010).

#### Plant Growth Promoting Activities

Considering that auxin production is one of the main characteristics of PGPB, we determined the biosynthesis of total indoles of Kd70 in a minimal saline medium with or without Trp (0.1 mg/ml). Results showed that strain Kd70 were able to produce high concentrations of indoles both in the presence (1.99 ± 0.07 μg/mg protein) and absence of Trp (1.30 ± 0.02 μg/mg protein). It is noteworthy that production of indoles by Kd70, in contrast with most others known PGPBs, is not significantly influenced by the presence of Trp in the growth medium, which implicates that Kd70 could possess both Trp-dependent and independent pathways for synthesis of indoles including IAA.

Kd70 was also found to be able to solubilize phosphorous on solid NBRIP culture medium by forming clear halos surrounding bacterial colonies. Results showed that Kd70 strain exhibit greater phosphate solubilizing activity (solubilizing index of 4.09) than *P. fluorescent* P21 (solubilizing index of 2.0), a previously isolated strain in our laboratory with known capacity to solubilize phosphor, used as positive control. In accordance with the genomic analysis, Kd70 was found to be positive for siderophore production as evidenced by the formation of clear orange halos surrounding bacterial colonies grown under iron-limiting conditions on CAS agar plates. When siderophore production was analyzed over time, we observed that yield of siderophore production (%Ys) for Kd70 increased during growth until reaching a maximum 7 days after incubation at 30°C (Supplementary Table S1). In contrast, siderophore-producing strain *P*. *syringae* 23, used as positive control, produced the highest levels of siderophores already after 3 days of growth. This result indicated that siderophore production of Kd70 seem to be directly correlated to cellular growth of this bacteria.

As the synthesis of siderophores has been reported to be strictly regulated by the presence of iron, we evaluated the ability of Kd70 strain to produce siderophores in solid CAS medium with and without the addition of iron. Supplementation with 50 μM FeCl_3_ resulted in a significant decrease in siderophore levels, while 100 μM of iron completely inhibited siderophore accumulation for both strains (Supplementary Figure S2).

#### Biofilm Formation Capacity of Kd70

Total biomass biofilm formation by strain Kd70 was determined using a colorimetric assay involving CV staining in a 96-well microtiter plate. Six days after bacterial inoculation, biofilm production by Kd70 was monitored and compared to biofilm formation by *P. fluorescent* 21, a strain with known capacity to form biofilm, and *B. pumilus*, strain Kd101, selected for poor biofilm formation. For all three strains tested the proportion of cells growing as biofilm was assessed relative to total bacterial growth. Results indicated that strain Kd70 showed a very good capacity to form biofilm on the surface of microtiter plates (Supplementary Figure S3), as exhibited by a greater capacity than the known biofilm-producing strain *P. fluorescent* 21.

#### Root Colonization and Growth Response of Sugarcane Plants Following Inoculation With Kd70

**FIGURE 1:**
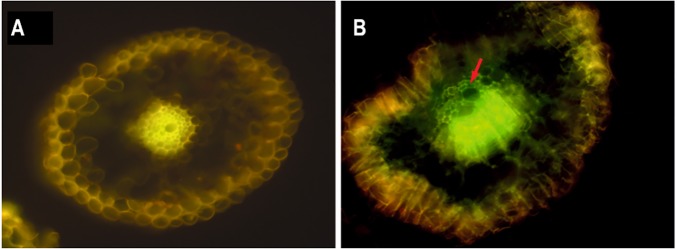
Epifluorescence microscopy of root internal tissue from greenhouse-grown sugarcane plants. **(A)** A hand-cut cross-section of the main root of a non-inoculated plant and **(B)**, a main root cross-section of a plant inoculated with *gfp*-tagged Kd70. The non-inoculated plant root is showing typical yellow auto-fluorescence of the vascular tissue which, is clearly differentiated from the strong green GFP fluorescence seen in the vascular vessels (arrow) and around the cortex cells in the *gfp-*Kd70 inoculated plant.

One-month-old *in vitro* micropropagated sugarcane plantlets challenged with a suspension culture of strain Kd70 harboring a constitutively expressed *gfp* gene (*gfp*-Kd70), demonstrated that this bacterial strain is able to colonize both vascular tissue and the parenchymal cortex in roots of sugarcane plants. Photos in **Figure 1** are showing cross-sections of roots of sugarcane plants non-inoculated (**Figure 1A**) or treated with *gfp-*Kd70 (**Figure 1B**). A clear green fluorescence is seen in the vascular tissue and around parenchymal cortex cells in the cross-section from *gfp-*Kd70-treated plants (**Figure 1B**) whereas non-inoculated plants only exhibited yellow autofluorescence of the vascular tissue (**Figure 1A**).

To evaluate the possible plant growth promoting activity of strain Kd70, the rhizosphere of 2-weeks-old sugarcane plantlets were inoculated with a Kd70 suspension and biomass development was compared with plants treated with water (mock-treatment) or a commercial bio-fertilizer product, Gramen (positive control). At the stage of tillering, no significant difference in the number of emerged plants among treatments were observed but plants inoculated with Kd70 exhibited a greater number of fully expanded green leaves in comparison with both, mock-treated and Gramen-treated controls (data not shown).

**FIGURE 2:**
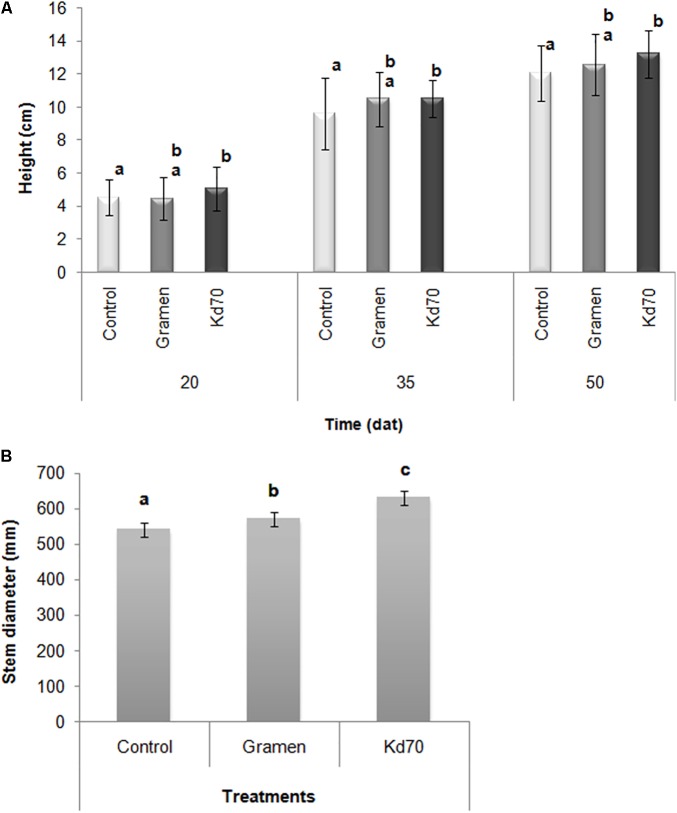
Plant growth-promoting effects of bacterial inoculation of greenhouse-grown sugarcane plants. Plants were treated with water (mock), commercial growth stimulating product AZ39 (Gramen) and strain Kd70. **(A)** Heights of plants were measured at 20, 35, and 50 days after treatment (dat) and **(B)**, Stem diameter of plants were measured 60 dat. Different letters indicate statistically significant differences when analyzed with LSD test (*P* ≤ 0.10).

The analysis of plant aerial growth showed that inoculation with Kd70 generated a significant increase in plant height across different sampling dates during the first 2 months of growth (**Figure 2A**). In addition, 60 days after treatment (dat) plants inoculated with either Gramen or strain Kd70 produced a significantly increased average stem diameter (**Figure 2B**) where the largest increase (16%) was found for plants inoculated with Kd70, as compared to mock-treated plants.

**FIGURE 3:**
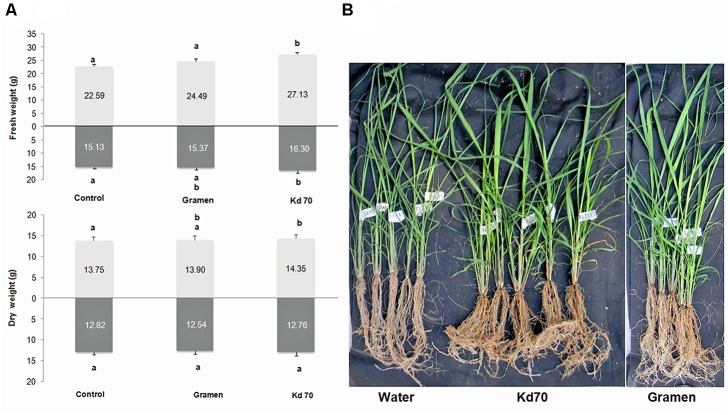
Effects of soil application of Kd70 and Gramen on the aerial and root biomass production in greenhouse-grown sugarcane plants. **(A)** Aerial and radical fresh and dry weight measurements for mock-, Gramen-, and Kd70-treated plants. Results are shown for plants evaluated 60 dat. Each value represents the mean of five replications and error bars indicate standard deviation. Different letters above and under bars indicate statistically significant differences at *P* ≤ 0.05. **(B)** Phenotypic response of sugarcane plants, variety LCP 85-384, treated with water (left), Kd70 (center), and Gramen (right). Photograph shows five representative plants 60 days after each treatment.

Data collected at the end of the experiment (60 dat) showed that inoculation with Kd70 led to a significant increase in both fresh (20%) and dry weight (4%) of aerial parts and on fresh weight of the radicular system (8%), when compared to mock-treated plants (**Figures 3A,B**). These increases in growth parameters were found to be substantially higher than those observed for Gramen-inoculated plants (8, 1.6, and 1%, respectively) (**Figure 3A**). However, when analyzing root biomass production the observed significant increase in fresh-weight was not accompanied by a corresponding increase in dry weight.

To demonstrate that Kd70 indeed had colonized roots of inoculated sugarcane plants and indirectly show that the plant growth stimulating effect could be attributed to this strain, plant extracts were obtained from macerated surface-sterilized roots from Kd70-inoculated and mock-inoculated plants. Four roots were macerated, two extracted from plants treated with Kd70 (K1 and K2), and two from mock-treated plants (W1 and W2) and tested for Kd70 colonization by PCR amplification using Kd70-specific primers for the cellulase encoding *GH3* gene. Only in the two samples from Kd70-treated plants was the amplification product corresponding to the *GH3* gene (269 bp) detected (Supplementary Figure S4A). In addition, extracts from the four roots (K1, K2, W1, and W2) were plated on agar saline medium with CMC as the sole carbon substrate. A total of 44 colonies from Kd70-treated and 20 from mock-treated sugarcane roots, able to grow on CMC plates, showed cellulase activity when tested with Congo Red (Supplementary Figure S4B). Supplementary Figure S4A shows the agarose gel electrophoresis of PCR amplification for 8 cellulase-positive colonies from Kd70-treated plants (K3–K10) and 7 colonies isolated from water-treated plants (W3–W9). The amplification product of 269 bp was only detected in 4 colonies isolated from extracts of Kd70-treated plant roots (Supplementary Figure S4A). This result was confirmed using a second pair of Kd70-specific primers amplifying a 251 bp specific DNA segment belonging to the *CBM6*-*CBM3* gene (data not shown).

#### Comparative Proteome Analysis

**FIGURE 4:**
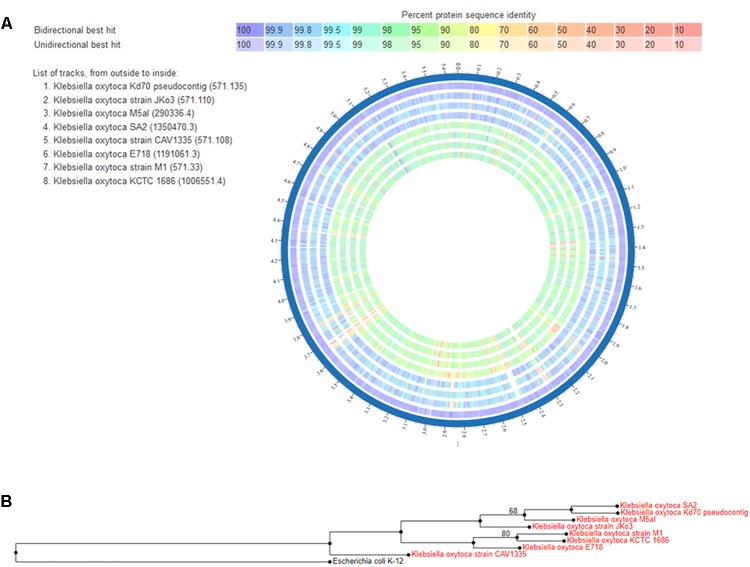
**(A)** Proteome comparison displayed by the circus tool at PATRIC. The entire proteome for each strain is aligned against the Kd70 proteome, used as reference strain. Track order from outside to inside is shown in the top left part of the figure where the reference strain, Kd70, is the most outside circle. The color key indicates the percentage of protein sequence similarity over the entire genome. **(B)** Phylogenetic tree of *Klebsiella* *michiganensis/oxytoca* isolates. Neighbor-joining tree based on whole genome of 8 *Klebsiella* isolates using the tree building application RAxML provided by the PATRIC platform. The tree was rooted using *E. coli* strain K-12.

The PATRIC (Gillespie et al., 2011) web resources were used for *in silico* comparison of the proteomes of *K*. *oxytoca/michiganensis* strains Kd70, JKo3, M5a1, SA2, CAV 1335, E718, M1, and KCTC 1686 (**Figure 4A**). Due to the very recent reassignment of several *K. oxytoca* isolates to *K. michiganensis*, the internal database of PATRIC has not yet been updated and all strains are therefore still named as *K*. *oxytoca*. The image in **Figure 4A** shows the percent identity across all the proteins in the comparison genomes compared to the Kd70, where strains JKo3 (human isolate) (Iwase et al., 2016), SA2 (endophyte sharing a natural niche with Kd70) (Mingyue et al., 2013), and M5a1 (soil isolate) (Bao et al., 2013; Yu et al., 2018) showed the highest degree of similarity with Kd70 over the entire proteome (blue/green color). The human pathogen strains CAV1335, E718, and KCTC 1686 together with environmental strain M1 showed a lesser similarity with Kd70 as visualized in the proteome comparison (green/yellow color) suggesting the existence of different genetic lineages within this species harboring both environmental and clinical isolates.

In accordance with the observation from the proteome comparison a phylogenetic study on the whole genome level showed a similar grouping and genetic relationship among these strains (**Figure 4B**). Three distinct groupings of the isolates included in the analysis were formed in the phylogenetic tree, where one genetic lineage included strains JKo3, M5a1, SA2, and Kd70 demonstrating their close relationship.

#### Genomic Island Predictions for Kd70

**FIGURE 5:**
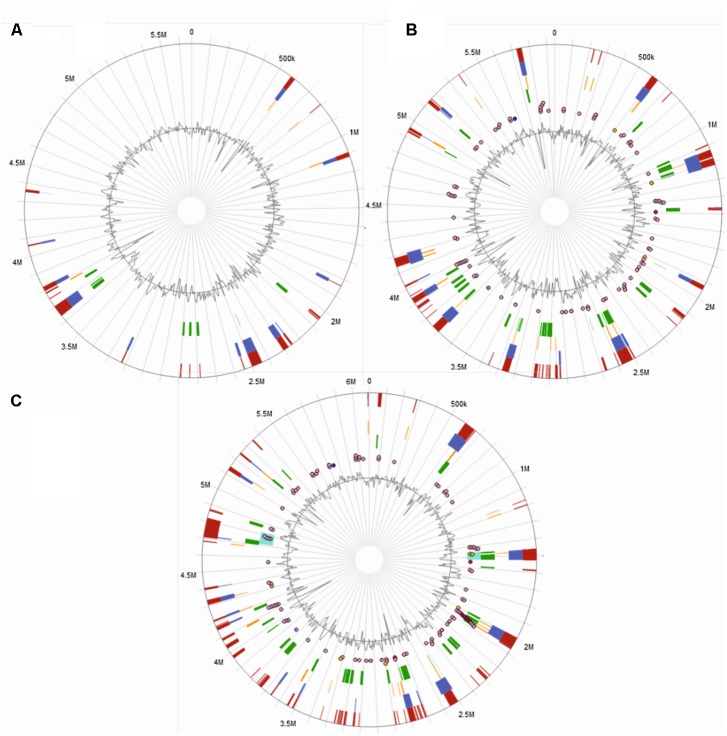
Prediction of genomic islands. IslandViewer 4 side-by-side views of GIs predictions for strain Kd70 **(A)**, JKo3 **(B)**, and E718 **(C)** genomes. The circle represents a single chromosome, with the outermost dark red bars indicating locations of all predicted GIs by integrating the four detection methods included in IslandViewer 4. Within the circle (starting from the outside toward the center), GIs predictions by software IslandPath-DIMOB are shown as blue, SIGI-HMM as orange, IslandPick as green and, Islander as turquoise bars. The putative virulence genes, antimicrobial resistance genes, and pathogen-associated genes are indicated as circular glyphs in the most interior of the circles.

Horizontal gene transfer is responsible for increased genome plasticity and is considered as an important factor in environmental adaptation and specification of microorganisms (Yoon et al., 2015; Bertelli et al., 2017). A major contribution to horizontal gene transfer in bacteria is through GIs, discrete DNA segments that in some cases are mobile and differ among closely related strains thus having a significant impact on the bacterial lifestyle including its pathogenicity. A subgroup of GIs that harbor one or more pathogen-associated genes (genes found in pathogens, but not in non-pathogens) is named pathogen associated islands (PAI). As many members of *K*. *oxytoca/michiganensis* are important human/animal pathogens we compared GIs of Kd70 with two clinical isolates (JKo3 and E718) to search for differences that could help understand the genetic background underlying differences in lifestyle between Kd70 and these two strains. The integrated interface for computational identification and visualization of GIs, IslandViewer 4, identified 26 putative GIs in the genome of Kd70 (**Figure 5A**). However, when analyzing the genomes of isolates JKo3 and E718 over three times more GIs were detected, 79 in JKo3 (**Figure 5B**) and 88 in E718, respectively (**Figure 5C**). Further analysis revealed that the genome of JKo3 harbored 6 and E718 26 genes encoding virulence factors associated to the detected GIs, including 3 (JKo3) and 4 (E718) pathogen-associated genes while the remaining corresponded to genes involved in antibiotic resistance. More importantly, no virulence factor was found to be associated to any of the putative GIs in the Kd70 genome. Interestingly, a very low coincidence were found in structure and genomic location of the GIs detected in the three strains, indicating an important difference in genome divergence among these strains due to horizontal genetic transfer, which at least partly helps explain their difference in lifestyle and pathogenicity.

#### Antibiotic Resistance

An important characteristic found in many members of *K. oxytoca/michiganensis* is multiple resistance to antibiotics, therefore a broad range antibiotic testing was performed for Kd70. The isolate displayed a moderate resistance to ampicillin but, was found to be susceptible to other β-lactam antibiotics including expanded-spectrum cephalosporins (Supplementary Table S2).

#### Ascending Urinary Tract Infection Assay in Mouse

The important differences in GIs and PAI content found between the genome of Kd70 and the two clinical isolates, JKo3 and E718, prompted us to test the pathogenicity of Kd70 *in vivo*. In an initial experiment a clinical strain of *K. oxytoca* isolated from a patient with cystitis was employed as positive control but due to the very high animal mortality rate, the uropathogenic *E. coli* 275 (UPEC 275) strain was selected as a model and positive control of ascending urinary tract infection in all further animal experiments.

**FIGURE 6:**
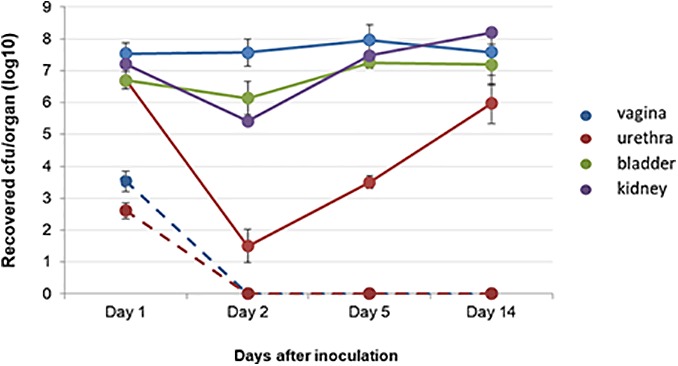
Kd70 is incapable of infecting the urinary tract in mice. Viable cells of virulent strain UPEC 275 (solid lines) and of Kd70 (dotted lines) recovered from vagina and organs of the urinary tract of inoculated BALB/c mice. Samples were taken from vagina washings (blue), urethra (red), bladder (green) and kidney (violet) homogenates. Number of viable bacteria shown is the mean of three-independent experiments.

BALB/c mice challenged with 5 × 10^6^ CFU UPEC 275, showed an increased bacterial colonization of both vagina and organs of the urinary tract throughout the sampling period, where viable bacterial cells were recovered in all exudates until the last day of sampling (**Figure 6**). In contrast, a rapid elimination, already within 2 days after infection, was observed for strain Kd70 in the murine vagina and urethra (**Figure 6**). Moreover, no viable Kd70 cells were ever detected in organ homogenates of the urinary tract during the study (data not shown).

## Discussion

The genomic analysis of Kd70 strongly indicated that this bacteria is indeed an endophytic strain with important plant growth promoting activity, although originally being isolated from the intestine of a larva of *D*. *saccharalis* (Dantur et al., 2015). This postulation was strongly supported by biochemical studies demonstrating auxin synthesis, phosphorus solubilization, siderophore production and biofilm formation, all activities which have been strongly related to PGPB (Glick, 2012; Ahemad and Kibret, 2014).

Eighty percent of all PGPB isolated from the rhizosphere have been shown to synthesize IAA (Patten and Glick, 1996; Khalid et al., 2004; Ahemad and Kibret, 2014), which is thought to help the plant getting access to soil nutrients by stimulating root development and root growth (El-Khawas and Adachi, 1999; Mishra and Kumar, 2015). Interestingly, IAA-synthesis for Kd70 was demonstrated not only when Trp was added but also in Trp-depleted growth medium, indicating a possible Trp-independent IAA synthesis pathway. However, no such pathway has been described yet in bacteria, although it has been suggested for many years (Prinsen et al., 1993).

Even more compelling evidence of an endophytic lifestyle for Kd70 was obtained from experiments of plant root colonization of sugarcane plants using *gfp*-labeled Kd70 cells. Kd70 readily colonized plant roots in a similar way that has been described for the two closely related isolates SA2 (An et al., 2001) and M5a1 (Yu et al., 2018). Furthermore, Kd70 demonstrated an important plant growth stimulating capacity of sugarcane plants as demonstrated by increased stem diameter and radicle development in greenhouse experiments.

It is a little surprising that no plant growth promoting activity has been reported for isolates SA2 and M5a1, although both have been shown to fixate nitrogen and colonize plant roots. Perhaps most interestingly though is the fact that JKo3, which was found to be closely genetically related to these three strains from plants and soil, comes from a clinical hospital collection in Japan (Iwase et al., 2016). There is no information available on the possible plant colonization capacity of this strain but it would be interesting to perform such a study as well as animal pathogenicity comparison studies among all four of these closely related strains.

Although there are numerous reports of strains of *K*. *michiganensis*/*oxytoca* isolated from different environmental habitats, and many recently published sequenced genomes of this species and others belonging to this genus, there are very few studies on whole genome comparisons among such isolates, especially between clinical and non-clinical strains, and it is therefore still an open question if a strain not isolated from an animal or human is capable of infecting mammals (Knittel et al., 1977; Konstantinidis et al., 2006; Mingyue et al., 2013). In an attempt to find genetic differences that could help explain the differences in ecological lifestyles within this species we compared the proteomic profile deduced from the genome sequences of several isolates from different ecological niches. A clear arrangement into genetic subgroups was observed and interestingly in at least two of these subgroups, both non-clinical and clinical isolates were found. This result suggests that an independent development of human/animal pathogenicity could have occurred within the two genetic lineages with a common origin of a plant/soil ancestor, a hypothesis supported by the existence of many genes involved in carbohydrate degradation and nitrogen fixation in clinical isolates and that clinical isolates are capable of colonizing environmental habitats.

When analyzing the Kd70 genome in more detail we found that it encoded members of most major viral factors associated with human pathogenicity, with the exception of type III fimbrae. However, with the important difference that the genome of Kd70 encodes significantly fewer members of such genes, than encountered in known human/animal pathogens of this species (Jiang et al., 2014). Moreover, when we compared GIs, virulence factors and putative PAIs with two clinical isolates of *K*. *oxytoca/michiganensis*, the closely related JKo3 and the genetically more distantly E718, important differences in genome organization was observed. First, no PAI-like regions were detected in Kd70 and the identified GIs were not shared between the human pathogens and Kd70. This observation, which indicated an impaired pathogenicity of Kd70, was confirmed in an ascending urinary tract infection study in mice that demonstrated a complete incapacity of Kd70 to infect these animals. This complete lack of animal pathogenicity is in contrast to the few studies on pathogenicity of environmental isolates of *Klebsiella* spp. conducted previously, although it is important to notice that all of these studies have been conducted in strains of *K*. *pneumoniae* and that there are almost no studies performed on isolates of *K*. *oxytoca/michiganensis* (Fouts et al., 2008; Huang et al., 2016).

Finally, the GIs and PAIs identified in the two human clinical isolates, JKo3 and E718, did not overlap between the two strains. This observation further supports the idea of a possible independent development of human pathogenicity among at least two subgroups of *K*. *oxytoca/michiganensis* as was suggested above. Moreover, these results strongly suggest that differences in pathogenicity and ecological lifestyle in this bacterial species to a large extent depends on horizontal gene transfer. Thus, Kd70 together with the other closely related members of this species should be excellent candidates for studying genes responsible for human and animal pathogenicity, horizontal gene transfer, plant growth promotion, and bacterial speciation. The very recent suggestion that strain M5a1 is representing a new species, closely related to *K*. *michiganensis* (Yu et al., 2018), indicate that a more detailed genetic analyze could indeed re-categorize several of these closely related isolates described as *K*. *michiganensis* or *K*. *oxytoca*.

This study clearly demonstrates the need to experimentally examine mammalian pathogenicity of bacterial isolates before discarding their potential usage in biotechnological applications. Kd70 is an excellent PGPB which could ultimately help developing a more efficient and sustainable production of both food and energy crops and our results indicate that this particular bacterial strain, belonging to a genus with important human and animal pathogens, could probably be employed in agricultural production without causing major health risks, although more studies are needed before such a conclusion definitely can be drawn.

## Author Contributions

KD and BW designed the study and wrote the manuscript. KD carried out and analyzed the bioinformatic studies, visualized experimental results, and prepared it for publication. NC, MC, and MT executed plant growth promoting experiments. CS and ÁJ conducted *in vivo* assays and antibiotic resistance test, respectively. NP supervised the *in vivo* assays. MB and AV generated the *gfp*-Kd70 strain. AC supervised the project, critically read the manuscript, and acquired funding. All authors contributed to data analysis and interpretation.

## Conflict of Interest Statement

The authors declare that the research was conducted in the absence of any commercial or financial relationships that could be construed as a potential conflict of interest.

## Footnotes

1 http://www.pathogenomics.sfu.ca/islandviewer/

2 http://www.cerela.org.ar
