# Supplementary material for: The Endophytic Strain Klebsiella michiganensis Kd70 Lacks Pathogenic Island-Like Regions in Its Genome and Is Incapable of Infecting the Urinary Tract in Mice
Source: Front Microbiol. 2018 Jul 16;9:1548. doi: 10.3389/fmicb.2018.01548 (PMC6054940; doi:10.3389/fmicb.2018.01548)
Supplement: Supplementary file 1 [file Table_1.doc]

**Table S1.** Time course of siderophore production evaluated on agar CAS plates.

*The yield of siderophore production (%Ys) was determined as [(halo diameter-colony diameter)/halo diameter] x 100. Mean values within the column followed by different letters are significantly different according to LSD test (P≤ 0.05).
