# Supplementary figures and images for: The Endophytic Strain Klebsiella michiganensis Kd70 Lacks Pathogenic Island-Like Regions in Its Genome and Is Incapable of Infecting the Urinary Tract in Mice
Source: Front Microbiol. 2018 Jul 16;9:1548. doi: 10.3389/fmicb.2018.01548 (PMC6054940; doi:10.3389/fmicb.2018.01548)

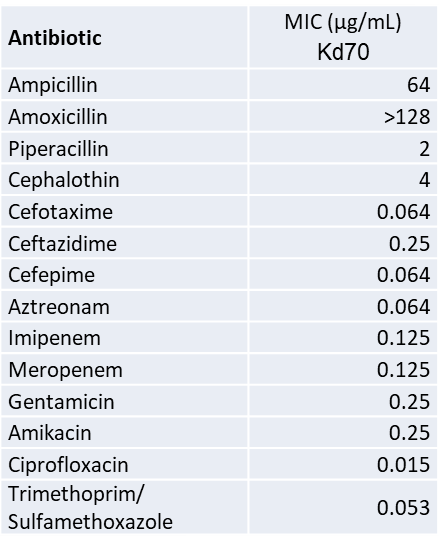
**Table S2.** Susceptibility of *K. oxytoca* Kd70 to common antibiotics.

Supplement: Supplementary file 2 [file Table_2.doc]
