# Supplementary material for: The Endophytic Strain Klebsiella michiganensis Kd70 Lacks Pathogenic Island-Like Regions in Its Genome and Is Incapable of Infecting the Urinary Tract in Mice
Source: Front Microbiol. 2018 Jul 16;9:1548. doi: 10.3389/fmicb.2018.01548 (PMC6054940; doi:10.3389/fmicb.2018.01548)
Supplement: Supplementary file 3 [file Image_1.pdf]

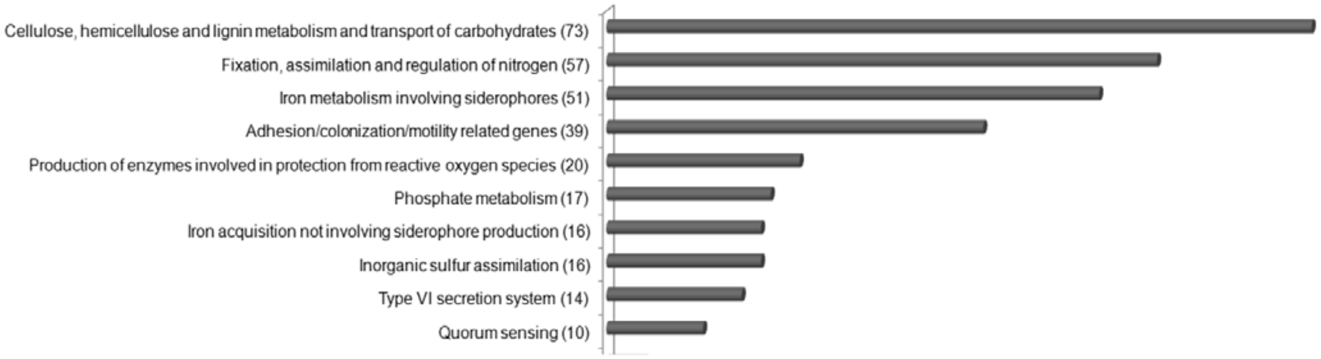

Figure S1| Genes involved in bacterium-plant interactions. A summary of genes identified in the Kd70 genome encoding proteins likely to be involved in bacterium-plant interactions. Numbers of genes corresponding to each class of proteins found in the Kd70 genome are shown in parenthesis.
