# Supplementary material for: The Endophytic Strain Klebsiella michiganensis Kd70 Lacks Pathogenic Island-Like Regions in Its Genome and Is Incapable of Infecting the Urinary Tract in Mice
Source: Front Microbiol. 2018 Jul 16;9:1548. doi: 10.3389/fmicb.2018.01548 (PMC6054940; doi:10.3389/fmicb.2018.01548)
Supplement: Supplementary file 4 [file Image_2.pdf]

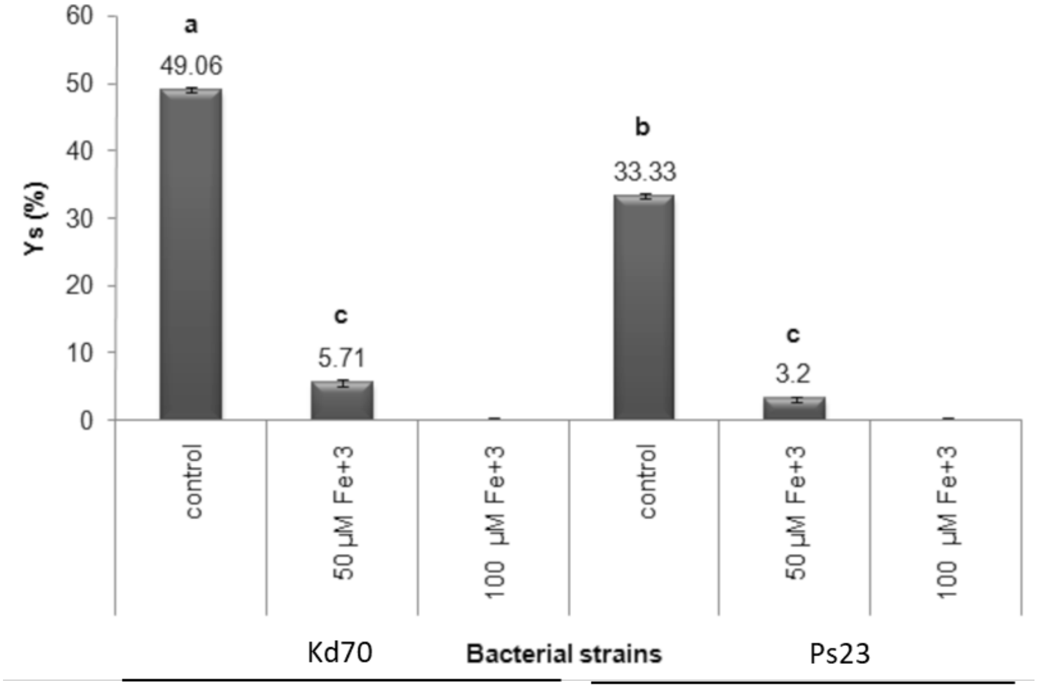

Figure S2| Effect of iron concentration on siderophore production by bacterial strains Kd70 and Ps23 using the CAS-agar plate assay. Data are means of three determinations and the error bars indicate SD. Different letters indicate significant differences at P = 0.05.
