# Supplementary material for: The Endophytic Strain Klebsiella michiganensis Kd70 Lacks Pathogenic Island-Like Regions in Its Genome and Is Incapable of Infecting the Urinary Tract in Mice
Source: Front Microbiol. 2018 Jul 16;9:1548. doi: 10.3389/fmicb.2018.01548 (PMC6054940; doi:10.3389/fmicb.2018.01548)
Supplement: Supplementary file 5 [file Image_3.pdf]

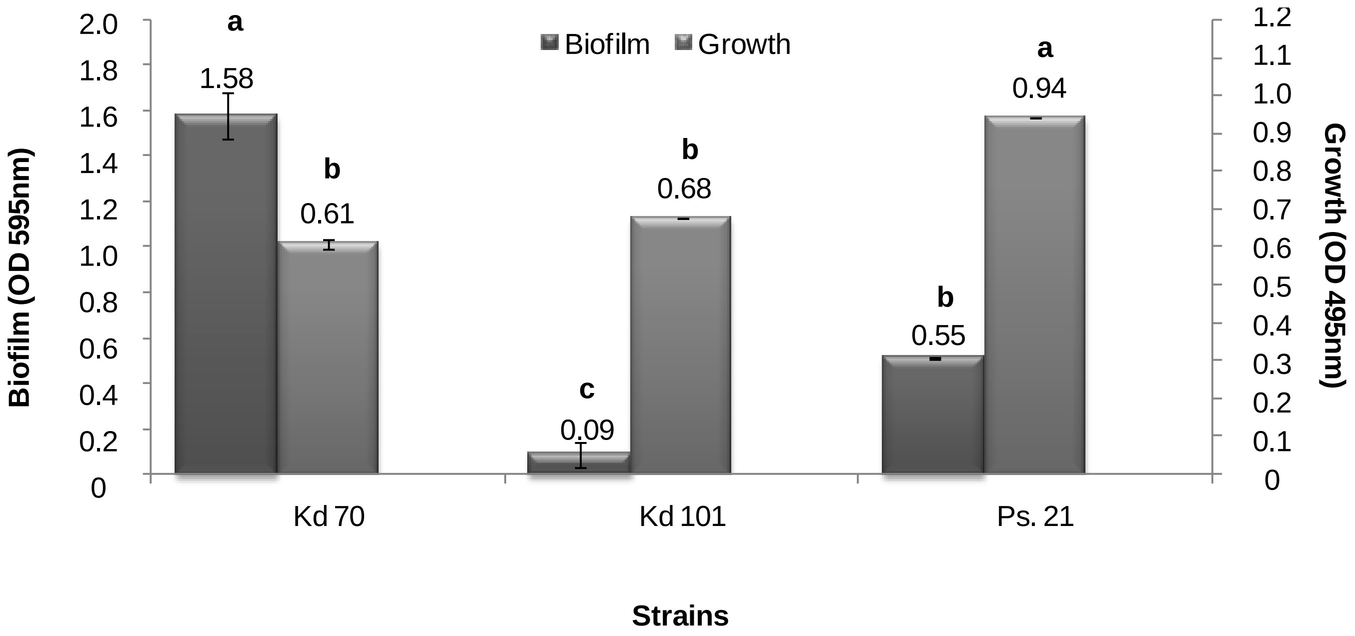

Figure S3 | Biofilm formation test. Biofilm formation efficiency relative to total bacterial growth of strain Kd70, in comparison to a control strain, *P. fluorescent* 21, selected for good capacity of biofilm production and Kd101 for poor biofilm formation.
