# Supplementary material for: The Endophytic Strain Klebsiella michiganensis Kd70 Lacks Pathogenic Island-Like Regions in Its Genome and Is Incapable of Infecting the Urinary Tract in Mice
Source: Front Microbiol. 2018 Jul 16;9:1548. doi: 10.3389/fmicb.2018.01548 (PMC6054940; doi:10.3389/fmicb.2018.01548)
Supplement: Supplementary file 6 [file Image_4.pdf]

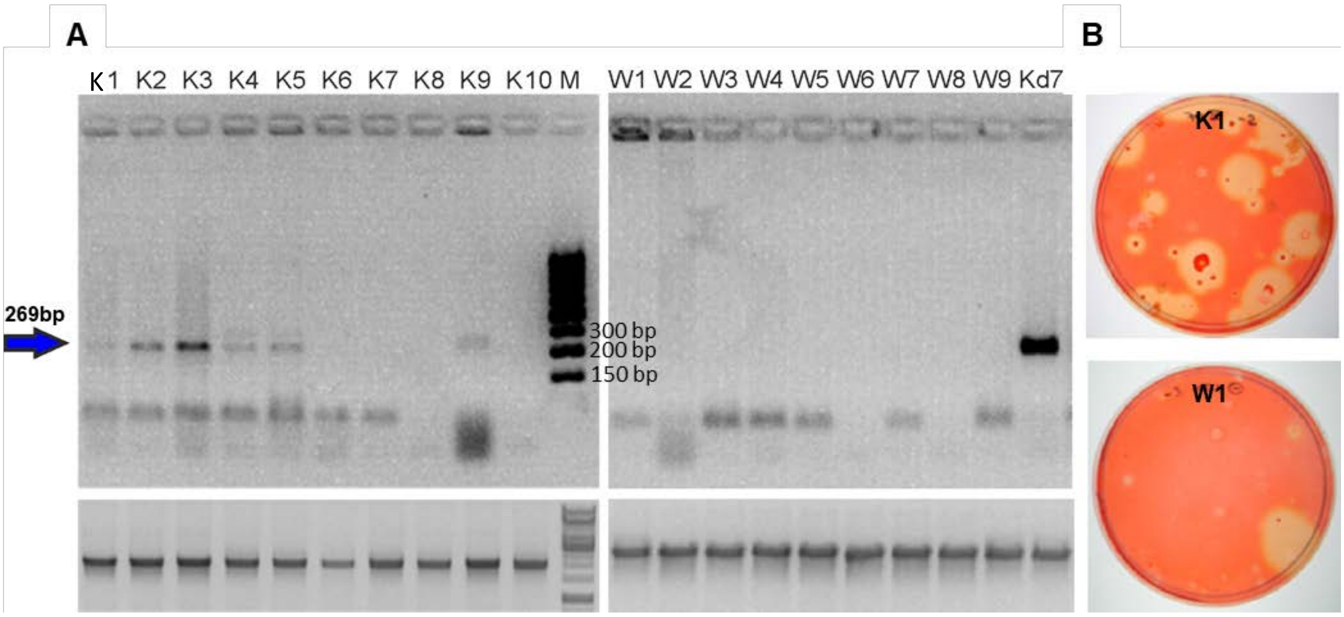

Figure S4| Detection of Kd70 in root plant tissue. (A) Agarose gel electrophoresis of PCR products (arrow) from total extracts of surface-disinfected sugarcane roots irrigated with *K. michiganensis* Kd70 (K1 and K2) and roots from mock-treated plants with no bacterial treatment (W1 and W2). Furthermore, PCR-amplification products from bacterial cellulose-degrading colonies, isolated by plating extracts from plants treated with Klebsiella (K3-K10) and mock-treated plants (W3-W9) are shown. Genome DNA isolated from Kd70 was used as an internal positive control of *GH3* amplification (Kd70) and amplification of 16S rDNA (lower part of the picture) was used as control of equal loading of total bacterial DNA. **(B)** Growth of isolated bacterial strains from roots of plants treated with Kd70 (K1) or water (W1) on Petri dishes containing CMC and later colored with Congo Red to visualize cellulolytic activity. A clear halo around bacterial colonies indicates cellulose degradation.
